# Noninvasive assessment of the common carotid artery hemodynamics with increasing exercise work rate using wave intensity analysis

**DOI:** 10.1152/ajpheart.00667.2017

**Published:** 2018-03-23

**Authors:** N. Pomella, E. N. Wilhelm, C. Kolyva, J. González-Alonso, M. Rakobowchuk, A. W. Khir

**Affiliations:** ^1^Institute of Environment, Health and Societies, Biomedical Engineering Research Theme, Brunel University London, Middlesex, United Kingdom; ^2^Centre for Human Performance, Exercise and Rehabilitation, College of Health and Life Sciences, Brunel University London, Middlesex, United Kingdom

**Keywords:** arterial distensibility, incremental exercise, ln*DU* loop, reflection index, wave speed

## Abstract

Noninvasively determined local wave speed (*c*) and wave intensity (WI) parameters provide insights into arterial stiffness and cardiac-vascular interactions in response to physiological perturbations. However, the effects of incremental exercise and subsequent recovery on *c* and WI have not been fully established. We examined the changes in *c* and WI parameters in the common carotid artery (CCA) during exercise and recovery in eight young, healthy male athletes. Ultrasound measurements of CCA diameter and blood flow velocity were acquired at rest, during five stages of incremental exercise (up to 70% maximum work rate), and throughout 1 h of recovery, and noninvasive WI analysis [diameter-velocity (*DU*) approach] was performed. During exercise, *c* increased (+136%), showing increased stiffness with work rate. All peak and area of forward compression, backward compression, and forward expansion waves increased during exercise (+452%, +700%, and +900%, respectively). However, WI reflection indexes and CCA resistance did not significantly change from rest to exercise. Furthermore, wave speed and the magnitude of all waves returned to baseline within 5 min of recovery, suggesting that the effects of exercise in the investigated parameters of young, healthy individuals were transient. In conclusion, incremental exercise was associated with an increase in local CCA stiffness and increases in all wave parameters, indicative of enhanced ventricular contractility and improved late-systolic blood flow deceleration.

**NEW & NOTEWORTHY** We examined hemodynamics of the common carotid artery using noninvasive application of wave intensity analysis during exercise and recovery. The hemodynamic adjustments to exercise were associated with increases in local common carotid artery stiffness and all waves’ parameters, with the latter indicating enhanced ventricular contractility and improved late systolic blood flow deceleration.

## INTRODUCTION

Wave intensity analysis (WIA) is a time-domain technique that provides surrogates of cardiac performance, vascular resistance, and the interaction of these parameters from blood pressure and blood flow velocity (*U*) measurements (26, 27). Typical WIA contours obtained in large arteries include three main peaks: forward- and backward-traveling compression waves (FCW and BCW, respectively) as well as a forward expansion wave (FEW). These waves provide information about left ventricular contractility in early systole (FCW) (23), left ventricular-induced deceleration of flow in late systole (FEW), and reflected waves returning toward the left ventricle in midsystole (BCW).

An important extension of the original WIA was the development of noninvasive WIA using direct and simultaneous measurements of diameter (*D*) and *U*, which can be acquired using Doppler ultrasound (8). This approach produces wave intensity curves that are almost identical to those produced using arterial pressure without relying on the calibration of pressure waveforms to brachial blood pressure and circumventing the problem of nonsimultaneous measurements using applanation tonometry. The temporally dispersed acquisition using applanation tonometry becomes especially erroneous when rapid physiological perturbations are involved. Another advantage of the ultrasound Doppler approach is the ln*DU* loop (8), often associated with the *DU* approach of WIA, which enables the noninvasive determination of local wave speed (c). This offers a useful tool to investigate local stiffness under different conditions, such as graded exercise, and enables the separation of the wave intensity waveform into forward and backward components (8).

Exercise is a complex and substantial physiological stress that involves alterations in autonomic nervous system activity, affecting cardiac dynamics (19, 36) and selectively altering peripheral vascular resistance (30). WIA is a unique tool with the potential to examine the cardiac-peripheral interaction during rapid vascular alterations induced by exercise (33); therefore, the aim of the present study was to examine WIA parameters measured at the common carotid artery (CCA) during incremental exercise work rates and throughout subsequent recovery. Specifically, we examined wave speed (*c*), as a surrogate marker of arterial stiffness, and the magnitude of incident and reflected waves as well as of reflection indexes to reveal the responses of the left ventricle and peripheral circulation. We hypothesized that indexes of left ventricular contractility and late systolic active blood flow deceleration would parallel increases in exercise work rate up to moderate to high work rates. We also predicted that measures of CCA resistance, defined as the ratio of mean pressure and mean blood flow, would correlate with values of wave intensity reflection indexes, representing the amount of energy that is reflected from the head circulation relative to the energy delivered into it.

## MATERIALS AND METHODS

### Study Group

Eight healthy endurance-trained men (triathletes and cyclists, age: 27 ± 4 yr, body mass: 68.1 ± 4.3 kg, height: 1.76 ± 0.1 m, body mass index: 22.0 ± 0.6 kg/m^2^) participated in the study. Participants completed a health questionnaire to possibly exclude those with overt cardiovascular or metabolic diseases, smokers, and participants taking any medication. None of the screened participants were excluded. The experimental procedures and potential risks were explained before testing, and written informed consent was obtained. The study was approved by the Brunel University London Research Ethics Committee and conformed with guidelines of the Declaration of Helsinki.

### Experimental Design

Participants attended the temperature-controlled (20–24°C) laboratory on two occasions separated by at least 48 h. A ramp incremental step exercise test to volitional fatigue was performed (29) using a semirecumbent cycling ergometer (Angio, Lode, Groningen, The Netherlands). The results obtained during this session were used to determine the individual maximal work rate (W_max_), defined as the power at which the participant was no longer able to maintain the required cadence (60 rpm).

In the second experimental session, participants rested for 30 min and then performed an incremental exercise trial based on their W_max_ and were monitored throughout 1 h of postexercise recovery. Hemodynamic measurements to determine carotid artery WIA were obtained throughout the experimental trial during this second session (see details in *Ultrasound Measurements*).

### Instrumentation

An SSD-5500 ultrasound system (Aloka, Tokyo, Japan) equipped with a 7.5-MHz linear array probe was used to insonate the right CCA, and a three-electrode ECG system (V5 configuration) was attached to the participant. Scans were performed longitudinally ~2 cm proximal to the bifurcation. The ultrasound echo tracking subsystem measured *D* with a resolution of 0.013 mm. The gates were positioned manually between the borders of media and intima of the superficial and deep walls and parallel to them. The *D* waveform was tracked automatically as the distance between the two gates over time obtained from the M-mode tracing. The pulse-wave Doppler subsystem measured *U* with a resolution of 0.012 m/s, and the Doppler gate was positioned manually in B-mode at the center of the vessel, parallel to the walls, ensuring that the insonation angle was always between 58 and 60°. Both *D* and *U* were simultaneously sampled at 1,000 Hz. Continuous noninvasive blood pressure was obtained using a photoplethysmography-based system from the left middle finger that was calibrated to oscillometrically measured brachial blood pressure (Finometer Pro, Finapres Medical Systems, Amsterdam, The Netherlands). The monitoring device estimated systolic blood pressure, diastolic blood pressure, and cardiac output using the model flow method (1,11). Mean arterial pressure (MAP) was calculated as MAP = one-third systolic blood pressure + two-thirds diastolic blood pressure. At high work rates, the finger plethysmorgraphy device was unable to reliably obtain a signal, and cuff pressures were recorded instead. The experimental exercise protocol was performed on the same semirecumbent cycling ergometer described above.

### Ultrasound Measurements

Measurements were acquired before exercise, during each stage of exercise, and at selected time points during postexercise recovery. *D* and *U* were obtained simultaneously and spanned 8−25 heart cycles, depending on the heart rate (HR) of the participant.

Participants arrived at the laboratory at least 2 h postprandial, having not performed strenuous exercise or consumed caffeine for 24 h and 12 h, respectively. They assumed a semirecumbent position (32°) and were asked to minimize head movement and maintain a stable position by holding the table handles, especially during exercise. Before any acquisition of carotid artery parameters, participants were instrumented and rested for 30 min. *D* and *U* were acquired twice during this period to determine baseline values. Participants then cycled continuously for 25 min, with work rate increasing every 5 min (20%, 40%, 60%, and 70% of each subject’s W_max_). *D* and *U* were acquired during the last 2 min of each exercise stage. After the incremental test, participants cycled for 30 s with no resistance and finally rested in the semirecumbent position throughout the next 60 min of recovery. Carotid imaging and wave recordings took place at 5, 10, 15, 20, 30, 40, 50, and 60 min postexercise. For every participant, two measurements were recorded at each time point.

### Data Analysis

Data were analyzed using custom designed algorithms in Matlab (version R2010b, The MathWorks, Natick, MA) based on previous work (15) and adapted for noninvasive determination of wave parameters (11). *D* and *U* waveforms were postprocessed using a Savitzky-Golay filter (32) with a second-degree polynomial and a 16-point half-width window to eliminate high-frequency noise.

For each measurement, at least three consecutive heart cycles presenting no obvious drift or dampening and retaining physiological features were selected for analysis; hemodynamic and wave parameters were calculated on a beat-by-beat basis for each selected segment and averaged to produce a single measure for each participant at each time point (working condition). To define a single control (baseline) condition, the four measurements taken at the preexercise stages (two at the 15-min time point and two at the 25-min time point for each participant) were averaged.

**Fig. 1.**
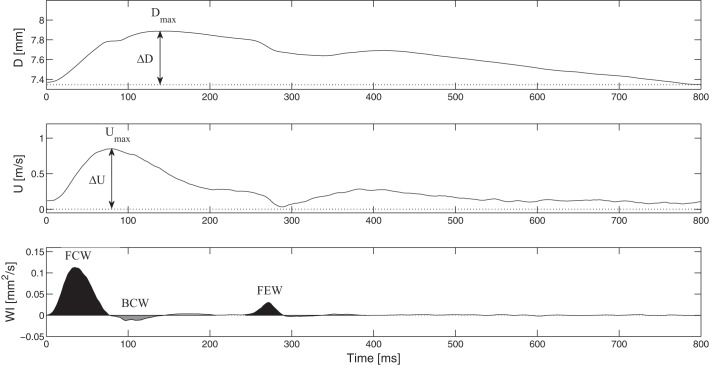
Example of waveforms. *Top*: carotid diameter (*D*) and blood flow velocity (*U*). The peak of the *D* waveform (*D*_max_), peak of the *U* waveform (*U*_max_), change in *D* or pulse (Δ*D*), and change in *U* or pulse (Δ*U*) are shown. *Bottom*: corresponding wave intensity. Black areas represent, from *left* to *right*, the forward-traveling compression wave (FCW) and forward expansion wave (FEW); the dark shaded areas represent the backward-traveling compression wave (BCW) and backward expansion wave (BEW), as reflections of FCW and FEW, respectively. The light shaded areas represent, from *left* to *right*, the reflections of BCW and BEW. Only FCW, BCW, and FEW are labeled.

The hemodynamic parameters extracted from *D* and *U* were *1*) peak (systolic) *D* (*D*_max_); *2*) change in *D* or pulse (Δ*D*), defined as the difference between peak (systolic) and trough (diastolic) values; *3*) peak (systolic) *U* (*U*_max_); *4*) change in *U* or pulse (Δ*U*), derived similar to pulse of *D* (see Fig. 1); and *5*) CCA resistance, defined as the ratio of MAP to CCA blood flow, where the flow was calculated as *U*·cross-sectional area of the vessel (0.25 π*D*^2^) and converted to ml/min. A circular CCA cross-section was assumed throughout the protocol.

**Fig. 2.**
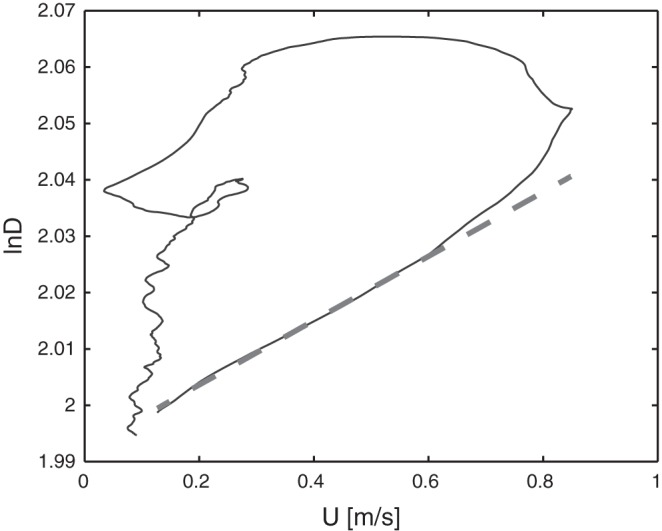
Example of a ln*DU* loop at rest for one participant. The regression line of the early systolic linear part is shown as the dashed line. *U*, blood flow velocity.

Considering that reflected waves were absent during the early systolic portion of each cardiac cycle, the slope of the linear part of ln*DU* loop (8, 14) was used to calculate *c* (in m/s) using the following equation and as shown in Fig. 2:

(1) $$c=\frac{1}{2}\frac{\text{d}U_{+}}{{\text{dln}D}_{+}}$$

where d*D* and d*U* are the incremental differences between adjacent elements of *D* and *U*, respectively. The subscript “+” indicates the forward components of the waves. Furthermore, carotid artery distensibility (*D*_s_) was calculated using *c* in the following Bramwell-Hill equation (6), as we have previously demonstrated (5):

(2) $$D_{\text{s}}=\rho^{-1}\times c^{-2}$$

where blood density (ρ) was assumed equal to 1,050 kg/m^3^.

The noninvasive wave intensity waveform (dI; in m^2^/s) was calculated as the product of d*D* and d*U* as dI = d*D* × d*U* and separated into forward and backward components (dI_+_ and dI_−_, respectively) by the following equation:

(3) $${\text{dI}}_{\pm }=\pm \frac{1}{4\left(D/2c\right)}{\left(\text{d}D\pm \frac{D}{2c}\text{d}U\right)}^{2}$$

The peak (in m^2^/s) and area (in m^2^) of the FCW, which is generated by the contraction of the left ventricle, were derived for each cardiac cycle from the amplitude and area under the early systolic peak observed in dI_+_, respectively (Fig. 1). Additionally, the peak and area of BCW, which is attributed to reflections from downstream circulation, were determined for each cardiac cycle from the amplitude and area, respectively, of the midsystolic peak present in dI_−_. Finally, the peak and area of FEW, which is generated by the decrease in shortening velocity of the left ventricle during late systole, were determined from the amplitude and area of the late systolic peak seen in dI_+_. The area parameters are associated with the energy of the waves, compared with similar parameters obtained with pressure-velocity WIA that have units of J/m^2^. Two different types of reflection indexes were computed: the modulus of the ratio of peak BCW to that of FCW (RI_I_), previously detailed by Borlotti et al. (5), and the modulus of the ratio of the energies of BCW and FCW (RI_E_). Mean values for all parameters were derived at each measurement among subjects. Table 1 shows definitions of all wave intensity parameters and the anatomic locations to which they refer.

**Table 1.** Definition of wave intensity parameters

Location Affected	Parameter Calculated	Physiological Indication
Left ventricle	FCW FEW	Ventricular contractility Late systolic flow deceleration
CCA	Wave speed	Distensibility
Downstream circulation	BCW |BCW/FCW|	Reflected wave Reflection index

BCW, backward compression wave; CCA, common carotid artery; FCW, forward compression wave; FEW, forward expansion wave.

### Statistical Analysis

All values are reported as means ± SD. Statistical analyses were performed using SPSS Statistics (v20, IBM, Armonk, NY). The mean values derived for each condition were tested via repeated-measures ANOVA, and significant effects were subsequently analyzed using Tukey’s post hoc tests. Statistical significance was assumed for *P* < 0.05. The reproducibility of the aforementioned hemodynamic and wave intensity parameters in young, healthy individuals has been established in a previous study (28). All parameters were reproducible both at rest and during exercise.

## RESULTS

### Hemodynamic Parameters

**Fig. 3.**
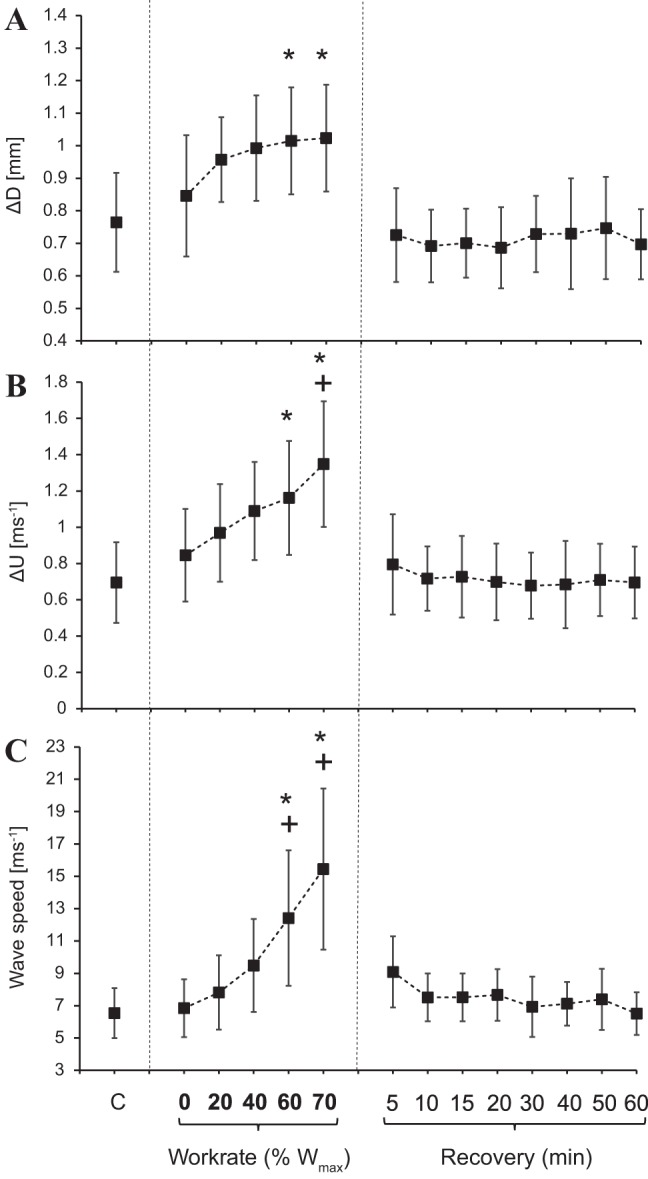
Means and SDs of the change in diameter or pulse (∆*D*; *A*), change in velocity or pulse (∆*U*; *B*), and wave speed (*C*) for each working condition of the protocol (*n* = 8). Vertical dashed lines separate control from exercise from recovery conditions. *Significant difference from control (*C*) and +unloaded cycling [0% individual maximum work rate (W_max_)], respectively.

Table 2 and Fig. 3, *A* and *B*, show the variation of hemodynamic parameters in different conditions. Δ*D* and Δ*U* increased during the later, more intense stages of exercise by up to 58% and 93%, respectively (*P* < 0.05), returning to baseline values within 5 min postexercise. *U*_max_ showed a similar pattern, increasing by 75% (*P* < 0.05) throughout exercise and decreasing back to values not different from baseline within 5 min of recovery. *D*_max_, however, did not change during incremental exercise.

**Table 2.** Hemodynamic parameters at selected work rates

Variable	Rest	40% W_max_	70% W_max_	5-min Recovery
Heart rate, beats/min	53 ± 5	120 ± 5*‡	164 ± 12*†	80 ± 10*
Systolic blood pressure, mmHg	125 ± 12	159 ± 19*	188 ± 15*†	123 ± 13
Diastolic blood pressure, mmHg	65 ± 5	73 ± 8	81 ± 9*	71 ± 10
Mean arterial pressure, mmHg	85 ± 6	102 ± 11	117 ± 11*†	88 ± 10
Distensibility, 10^−5^ Pa^−1^	2.6 ± 0.9	1.4 ± 1.1	0.6 ± 0.4*†	1.4 ± 0.9
Cardiac output, l/min	5.8 ± 0.5	15.1 ± 2.2*†	21.9 ± 5.7*†	8.0 ± 0.6
Peak of the diameter waveform, mm	7.3 ± 0.6	7.8 ± 0.4	8.1 ± 0.6	7.3 ± 0.5
Peak of the velocity waveform, m/s	0.8 ± 0.2	1.1 ± 0.3	1.4 ± 0.4*	0.8 ± 0.3
Common carotid artery flow, ml/min	454 ± 146	519 ± 192	667 ± 234*	405 ± 241

Data are means ± SD; *n* = 8. W_max_, maximum work rate.

* Significantly different from control (rest);

† significantly different from unloaded cycling (0% W_max_);

‡ Significantly different from the previous exercise condition.

### Wave Speed and Distensibility

**Fig. 5.**
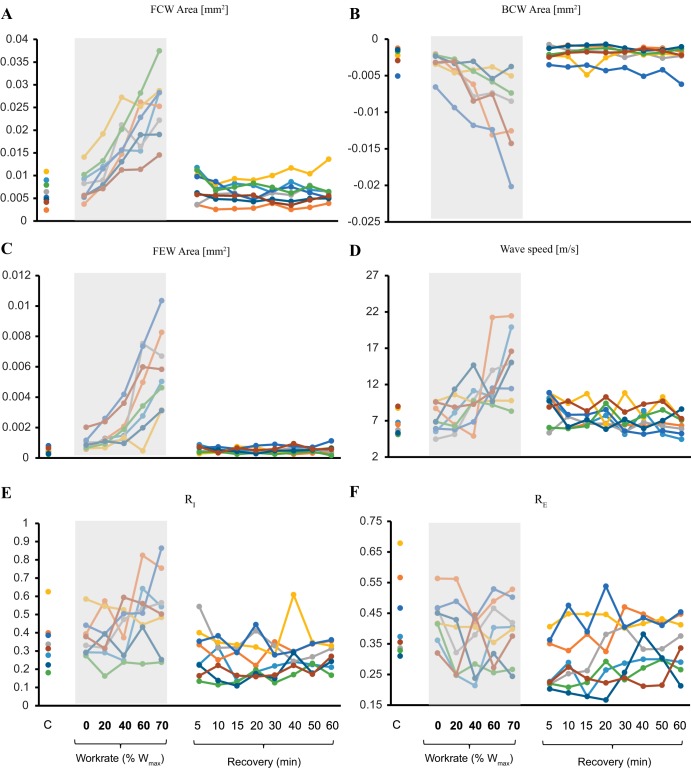
Individual responses of forward-traveling compression wave (FCW) area (*A*), backward-traveling compression wave (BCW) area (*B*), forward expansion wave (FEW) area (*C*), wave speed (*c*; *D*), ratio of peak BCW to that of FCW (RI_I_; *E*), and ratio of the energies of BCW and FCW (RI_E_; *F*) for all working conditions (*n* = 8). The shaded areas define the exercise period, separating rest from recovery conditions. W_max_, maximum work rate.

Changes in *c* during incremental exercise are shown in Fig. 3*C*, and distensibility values are shown in Table 2. Individual values are shown in Fig. 5*D*. On average, *c* significantly increased during incremental exercise by up to 136% compared with baseline (*P* < 0.05) and returned to values not different from baseline within 5 min of recovery. At the individual level, one subject’s response didn’t show any increase during exercise and was characterized by consistent fluctuations in recovery. *D*_s_, by definition, followed an inverse pattern, decreasing during incremental exercise by as much as 79% (*P* < 0.05) and then slowly stabilizing near baseline values in recovery.

### Wave Intensity Parameters

**Fig. 4.**
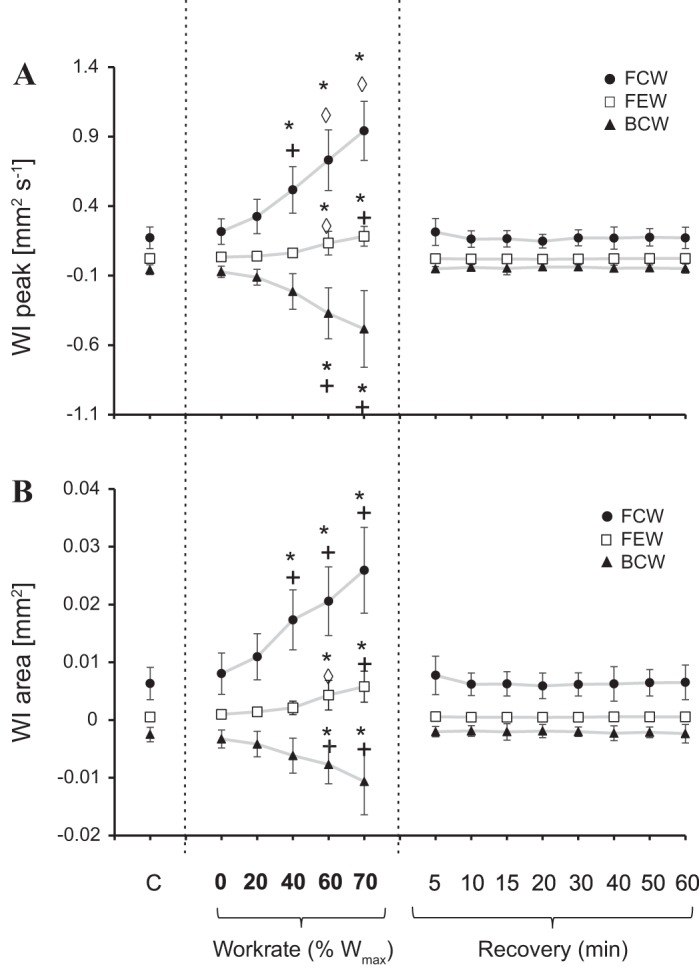
Means and SDs of wave intensity (WI) peaks (*A*) and WI areas (*B*) for each working condition of the protocol (*n* = 8). Vertical dashed lines separate control from exercise from recovery conditions. *Significant difference from control (*C*), +unloaded cycling [0% individual maximum work rate (W_max_)], and ◊previous condition, respectively. *c*, wave speed; FCW, forward-traveling compression wave; FEW, forward expansion wave; BCW, backward-traveling compression wave.

Wave intensity parameters are shown in Figs. 4 and 5, *A*–*C*. The peak and area of the FCW followed similar trends, with values continuously increasing during incremental exercise, up to 452% and 316% (*P* < 0.05) on average, in relation to baseline values, respectively, then returning to values not different from baseline within 5 min of recovery.

**Fig. 6.**
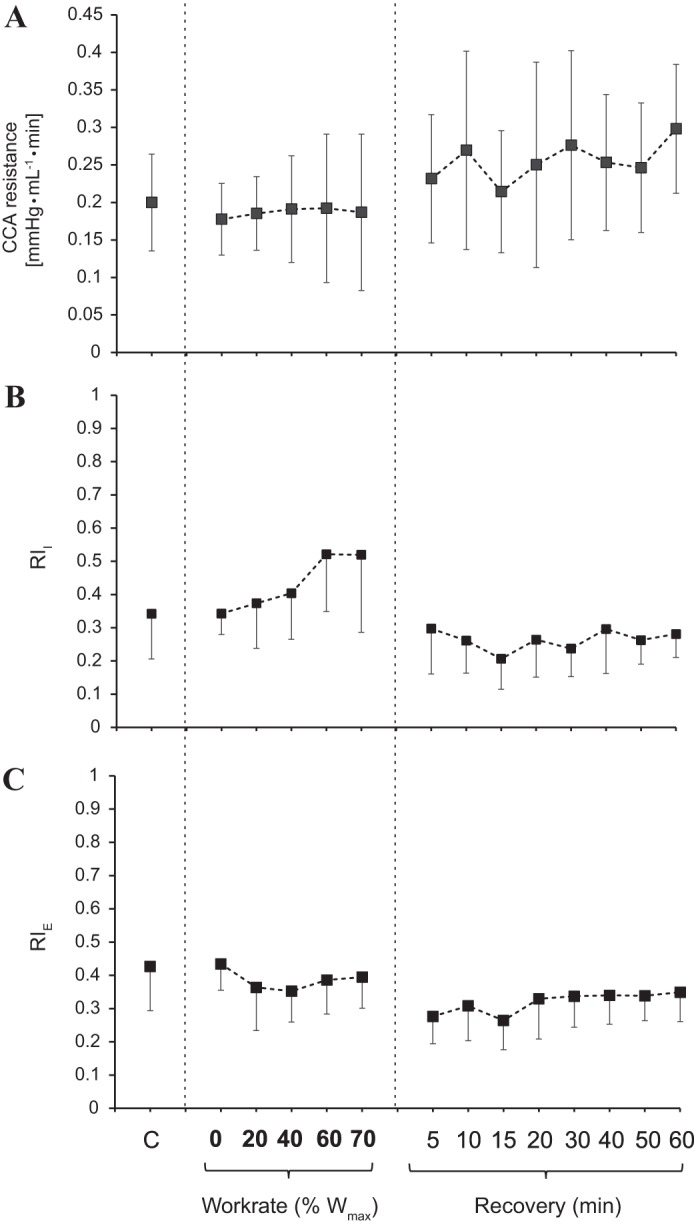
Means and SDs of common carotid artery (CCA) resistance (*A*), ratio of peak backward-traveling compression wave to that of forward-traveling compression wave (RI_I_; *B*), and ratio of the energies of backward-traveling compression wave and forward-traveling compression wave (RI_E_; *C*) for each working condition of the protocol (*n* = 8). Vertical dashed lines separate control from exercise from recovery conditions. *c*, local wave speed; W_max_, maximum work rate.

The peak and area of the BCW followed an inverse trend compared with FCW variables. These reductions with exercise were up to 700% and 390% (*P* < 0.05) on average for peak and area values, respectively, and then quickly returned to baseline during recovery (within 5 min postexercise). At the individual level, one subject’s BCW response did not show any increase during exercise and was characterized by consistent fluctuations in recovery. The peak and area of the FEW followed a similar pattern to that of FCW but with a much greater increase during exercise on average, reaching 718% and 900% above baseline (both *P* < 0.05), respectively, and returning toward control values within 5 min of recovery. Finally, reflection indexes and CCA resistance are shown in Figs. 5, *E* and *F*, and 6. RI_I_, RI_E_, and CCA resistance did not change throughout the protocol.

## DISCUSSION

In this study, we determined local wave intensity parameters and *c* noninvasively at the CCA at rest and during incremental exercise up to 70% W_max_ and subsequent recovery. Our technique combines direct and simultaneous measurements of *D* and *U* and does not require blood pressure, which is a distinct advantage because reliable noninvasive blood pressure waveforms during exercise are difficult to obtain and often involve invasive catheterization. The main findings were that *c* in the CCA, which provides a measure of arterial stiffness, increased with increasing work rate yet returned to baseline values rapidly within 5 min of recovery. Wave intensity parameters (FCW, BCW, and FEW peaks and energies) increased during moderate and heavy exercise, again rapidly returning to baseline values within 5 min of recovery. However, reflection indexes and CCA resistance remained unaltered, suggesting that the energy reflected centripetally and measured at CCA increased proportionally with the energy delivered by the heart during graded exercise.

### Distensibility and Wave Speed With Increasing Exercise Work Rate

Distensibility of carotid arteries has wide clinical acceptance as a prognostic indicator of hypertension and cardiovascular disease (21). It represents a dynamic characteristic of the vessel, which decreases with increasing pressure and *D*, and strongly influences pulsatility (16). The ln*DU* loop offers a tool to directly evaluate local distensibility or stiffness by first measuring *D* and *U* and then using the Bramwell-Hill equation to compute these values (5). In our study, we measured distensibility over a wide range of carotid diameters and found very good agreement between our distensibility data and previous work (21), at rest, for a similar but nontrained cohort. Interestingly, our *c* values measured in a single vessel are quantitatively similar to previous studies (35) that determined *c* over the whole aorta of a young, healthy, but untrained cohort, suggesting a single point carotid measure may be worth exploring as a whole aorta surrogate.

An important finding was the increase in stiffness and its relationship with exercise work rate up to 70% W_max_. Mechanistically, this likely relates to sympathetic activity and pressure-related distention of the artery. Changes in blood flow and vascular resistance during incremental exercise occur regionally, depending on the active or inactive status of the tissues and organs (9), and previous work supports the idea that the muscle mass involved in the exercise explains part of the sympathetic response, impacting the magnitude of CCA smooth muscle contraction and stiffness (34). In addition, work by Victor et al. (40) suggests around a two- to threefold potentiated muscle sympathetic nerve activity with large muscle mass dynamic exercise compared with small muscle mass handgrip exercise. HR, per se, does not affect pulse wave velocity or MAP (19, 41), although the increase in pulse wave velocity might be secondary to an increase in MAP. Using the ln*DU* loop method and selecting the initial phase of systole to derive *c* ensured that HR was not confounding the measurement directly. The apparent stiffening of the vessel likely involves the gradual recruitment of collagen fibers as the vessel distends with increasing arterial pressure (20, 35). Interestingly, *c* quickly returned to values not different from baseline in recovery, a result in agreement with the central vascular alterations occurring in athletes during recovery, as highlighted by Müller et al. (18). This may relate to a rapid arterial pressure drop concomitant to a reduction in sympathetic nervous system activity.

### Wave Intensity Adjustments to Incremental Exercise

#### Forward and backward compression waves and reflection indexes.

As exercise work rate increased up to 70% W_max,_ there was a progressive increase in the energy delivered by the heart, judging by both greater peaks and areas of FCW. Specifically, wave intensity is a measure of the energy flux carried by the waves, and it is generally much less than the total kinetic and potential energy associated with the waves (27); nonetheless, it is useful for the estimation of cardiovascular performance. Similar to pressure-velocity wave intensity, *DU* wave intensity FCW can be used to quantify the changes (enhancement in this study) of cardiac contractility.

Simultaneous to the increased contractility, the energy reflected back into the CCA increased almost proportionately. Similarly, Babcock et al. (2) noted increases in both FCW and BCW after a brief maximal 30-s exercise bout. However, isolated examination of the BCW does not provide adequate information to properly assess downstream vascular resistance, and CCA wave intensity reflection indexes, such as RI_I_ and RI_E_, are needed for these estimates.

The CCA bifurcates into the internal carotid artery, supplying the brain, the external carotid artery, and other head tissues, such as facial tissues. The internal carotid artery supplies much of the global cerebral blood flow, together with the vertebral artery (31). Bleasdale et al. (4), using CCA WIA, demonstrated a reduction in the reflection index with CO_2_ administration and suggested reduced cerebrovascular resistance (CvR). However, Sato et al. (31) reported that vascular resistance in various head conduit arteries is differentially altered during incremental exercise, in relation to the complex interaction of thermoregulatory adjustments in the facial vasculature and alterations in CvR. These divergent responses in cerebral and extracranial circulations combine to alter reflected waves arriving at the CCA. Therefore, reflections from different microcirculatory beds in the head, such as the cerebral and the skin microcirculation, cannot be differentiated by CCA WIA exclusively during exercise.

Based on the patterns exhibited by the reflection indexes in our study, there wasn’t any change in the ratio of magnitude of reflected to incident waves within the examined exercise work rates in association with the absence of changes in CCA resistance. This is likely due to a simultaneous change in vascular resistance within the facial circulation, combined with changes in the opposite direction in CvR, to modulate arterial inflow with enhanced perfusion pressure. Specifically, it was found that CvR normally decreases up to moderate work rates (~60% of maximal aerobic power) and increases thereafter as the work rate rises to maximal levels (10, 13, 15, 34, 38), a unique response compared with locomotor muscle tissues (17).

#### Forward expansion wave.

The systolic contraction and diastolic relaxation of the ventricle are controlled by the myofibrillar arrangement within the wall, which dictates the direction and degree of the twisting and untwisting of the chamber. Because of the twisting mechanics, crucial for the optimal performance of the heart (3, 22, 37), major and minor axes of the left ventricle reach peak shortening velocities at different times during systole, and the overall “suction” effect acts as a breaking force (25, 35). It is believed that FEW rises when the major axis begins decelerating after having reached its peak velocity; at this stage, the minor axis is already in its decelerating phase (24). The FEW is then supported by a late phase of ventricular untwisting before the aortic valve closes.

Previous work by Babcock et al. (2) involving prolonged high work rate cycling observed reduced FEW magnitudes in early recovery from prolonged intense exercise, suggesting dysfunction. In our study, however, FEW peak and area increased during all stages of incremental exercise up to 70% W_max_, suggesting that the left ventricle, while contracting with increasing force, is also able to decelerate more rapidly. We believe that the characteristics of the exercise performed, namely, cycling briefly at high work rates in Babcock et al.’s study compared with longer durations at high work rates in our study, explain these contrasting findings. As such, the incremental exercise may have improved deceleration mechanics, leading to more efficient relaxation, which would enhance diastolic filling duration. This enhanced diastolic relaxation would reduce ventricular pressure more rapidly, thus facilitating passive filling of the ventricles in early diastole.

### CCA Blood Flow Responses to Incremental Exercise

Hellstrom et al. (12) suggested that both *D* and *U* play an important role for CCA blood flow. However, there were no significant changes in carotid *D* in a study by Sato et al. (31) during graded exercise. Our findings agree with Sato et al., indicating that increases in velocity account fully for the increases in CCA blood flow during incremental exercise. Increases in *U*_max_ and ∆*U* between control and the heaviest work rate were 74% and 94%, respectively, although the increase in ∆*D* was 34% and *D*_max_ did not change.

Further considerations about the nature of the blood flow can be elucidated from Womersley and Reynolds numbers. During exercise, frequency of waves and blood viscosity significantly change, affecting the Womersley number and velocity profile. The frequency of oscillations was derived from HR, ρ was assumed to be constant, the mean radius was measured, and blood viscosity was assumed to linearly increase up to 15% at W_max_, a value obtained by averaging results of Connes et al. (7), who determined the increases in blood viscosity in athletes with different levels of hemoglobin saturation. During recovery, blood viscosity was assumed to fall to baseline values (39). The Womersley number almost doubled, from 4.4 at baseline value to 8.1 at 70% W_max_. Therefore, the velocity profile in the CCA at rest and during recovery could be considered parabolic, whereas it became prominently flat during exercise. A similar calculation, using *D*_max_ and *U*_max_, leads to values of the Reynolds number ranging from 1,693 ± 470 at baseline to 2,946 ± 733 at 70% W_max_. Values postexercise were similar to baseline (the average among all measurements in recovery being 1,703 ± 526). Thus, the flow became turbulent and inertial forces more prominent during exercise compared with adjusted to control values within 5 min of the recovery period.

The nature of the flow can pose real challenges to the measurements during graded exercise because turbulence and nonparabolic velocity profile occur.

### Limitations and Methodological Considerations

Examination of the test power curve for RI_E_ using *t*-distribution and hypothesized RI_E_ means found throughout the protocol showed the test power values were generally low, especially during exercise. Specifically, the power of the statistical analysis was between 0.2 and 0.4 during exercise and between 0.5 and 0.9 during recovery; therefore, the probability of error was between 0.6 and 0.8 during exercise and between 0.5 and 0.1 during recovery. This is because of the sample size, consisting of eight subjects. Therefore, the interpretation of the findings needs to be treated with caution. Nonetheless, some of our results were associated with statistical significance, and individual values of relevant parameters were displayed to better explain general trends. Further studies evaluating wave intensity patterns and reflection indexes in the internal and external carotid arteries and the vertebral arteries are warranted to specifically assess the cerebral vascular resistance during graded exercise.

### Conclusions

Using completely noninvasive methods involving Doppler and imaging ultrasounds of the CCA during incremental exercise and subsequent recovery, we obtained wave intensity parameters that provided insights into cardiac dynamics and the local vascular distensibility in athletes. As exercise work rate increases, carotid artery stiffness rises. Moreover, the peak magnitude and area of the FCW increase with exercise work rate, indicating enhanced ventricular contractility, which also increases the magnitude of the BCW. The reflection indexes and CCA resistance do not change with exercise, highlighting that the increased magnitude of reflections is more likely due to the enhanced contractility rather than changes in vascular resistance, at least at the CCA. The increase in FEW during exercise suggests an improvement in left ventricular active deceleration of blood flow in late systole, potentially improving filling time during diastole. Within 5 min of recovery, the magnitude of all waves returned to baseline and remained unchanged for 1 h, suggesting that effects of incremental exercise in the investigated hemodynamic and wave intensity parameters of young, healthy individuals are transient. These hemodynamic adjustments to exercise are associated with enhanced ventricular contractility and improved late systolic blood flow deceleration.

## DISCLOSURES

No conflicts of interest, financial or otherwise, are declared by the authors.

## AUTHOR CONTRIBUTIONS

C.K., J.G.-A., M.R., and A.W.K. conceived and designed research; N.P. and E.N.W. performed experiments; N.P. and E.N.W. analyzed data; N.P., E.N.W., C.K., J.G.-A., M.R., and A.W.K. interpreted results of experiments; N.P. and E.N.W. prepared figures; N.P., C.K., and M.R. drafted manuscript; N.P., E.N.W., C.K., J.G.-A., M.R., and A.W.K. edited and revised manuscript; N.P., E.N.W., C.K., J.G.-A., M.R., and A.W.K. approved final version of manuscript.
